# Analysis of Monosodium l-Glutamate in Food Products by High-Performance Thin Layer Chromatography

**DOI:** 10.4103/0975-1483.66795

**Published:** 2010

**Authors:** Veni N Krishna, D Karthika, Devi M Surya, MF Rubini, M Vishalini, YJ Pradeepa

**Affiliations:** *Department of Pharmaceutical Analysis, J. S. S. College of Pharmacy (Off Campus College of J. S. S. University, Mysore), Rocklands, Ooty - 643 001, Tamilnadu, India*

**Keywords:** Food products, HPTLC, monosodium L glutamate

## Abstract

A simple, fast, specific, and precise high-performance thin layer chromatography method has been developed for the estimation of monosodium l-glutamate (MSG) in food products. Aluminum plates precoated with silica gel 60 GF_254_were used as stationary phase and a mixture of methanol–chloroform–formic acid in the ratio 5:5:1 (v/v) as mobile phase. Quantification was carried out by postchromatographic derivatization using 1% ninhydrin solution, and the developed spots were scanned by using a densitometer in absorbance mode at 485 nM. The R_f_value of MSG was 0.64. The results of the analysis have been validated statistically and by the recovery studies. Linearity was observed in the concentration range of 400–1000 nG.

## INTRODUCTION

Monosodium L-glutamate (MSG), chemically known as 2-amino pentane dioic or 2-amino glutaric acid, is normally used as a flavor-enhancing ingredient more commonly used in traditional Asian cuisine. This stimulates specific receptors located in taste buds such as the amino acid receptor T_1_R_1_/T_1_R_3_or other glutamate receptors like the metabotropic receptors, which induce the taste known as umami. Only l-glutamate enantiomer has the flavor-enhancing properties. In 1959, the Food and Drug Administration (FDA) classified monosodium glutamate as a “generally recognized as safe” substance. But in 1995, FDA Commissioned report acknowledged that an unknown percentage of the population may react to monosodium glutamate and develop monosodium glutamate symptom complex, a condition characterized by the following symptoms: headache, nausea, rapid heartbeat, bronchospasm, chest pain, drowsiness, weakness, and sweating.[1] Monosodium glutamate is absorbed very quickly in the gastrointestinal tract, so MSG could spike blood plasma levels of glutamate.[2] This is in a class of chemicals known as excitotoxins. High levels of which have been shown in animal studies to cause damage to areas of brain unprotected by the blood–brain barrier and that a variety of chronic diseases can arise out of this neurotoxicity.[3,4] Spectrophotometric,[5,6] derivative HPLC,[7] HPLC with UV detection, fluorescence detection,[8,9] GC,[10] paper chromatography,[11] and potentiometric methods[12] were reported for analysis of MSG in food products. In this paper, we report a new, rapid, sensitive, precise, and selective HPTLC method for the determination of MSG in food products.

## MATERIALS AND METHODS

### Solvents and chemicals

Monosodium l-glutamate was procured from Sigma-Aldrich limited, India. Food products were procured commercially. Chromatographic grade solvents such as methanol, chloroform, acetone, and formic acid were obtained from Qualigens Chemicals, Mumbai, India. Ninhydrin was procured from Rankem Chemicals, Mumbai, India.

### Standard and sample solutions

Monosodium l-glutamate (100 mg) was accurately weighed into a 100-mL volumetric flask, dissolved in water, and the solution was diluted to volume with the same solvent to furnish a working standard.

Accurately weighed sample equivalent to 1 g and transferred to a 100-mL volumetric flask, dissolved in water (50 mL), sonicated for 15 min in an ultrasonicator, and made up to the volume with the same solvent. The solution was then filtered through Whatmann’s No. 42 filter paper. One milliliter of the filtrate was taken in a 10-mL volumetric flask and diluted to the volume with methanol and used for analysis.

### Chromatography

Chromatography was performed on aluminum-backed silica gel 60 GF_254_TLC plates prewashed with methanol.

Standard solutions of MSG were transferred to different 10 mL volumetric flasks and diluted to volume with the methanol such that the final concentrations were 0.4–1 μg/μL. Standards and three different sample solutions were applied to the TLC plates as 8 mM bands with 9 mM space between two bands using a Camag Linomat IV sample applicator. Plates were developed with a mobile phase of methanol–chloroform–formic acid 5 + 5 + 1 (v/v) in a TLC twin trough chamber.

After development, the plates were derivatized with 1% ninhydrin solution in acetone and dried at 60°C for 5 min. The quantification of the standards and samples were performed by means of a Camag TLC scanner III controlled by WinCATS 4.06 version software. The amount of MSG in the sample solutions were computed from the calibration plot.

## RESULTS AND DISCUSSION

### Chromatography

**Figure 1:**
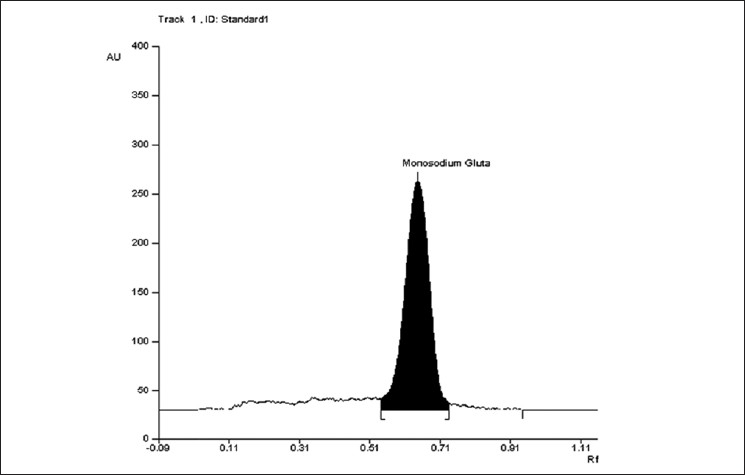
Typical HPTLC chromatogram of standard monosodium glutamate

**Figure 2:**
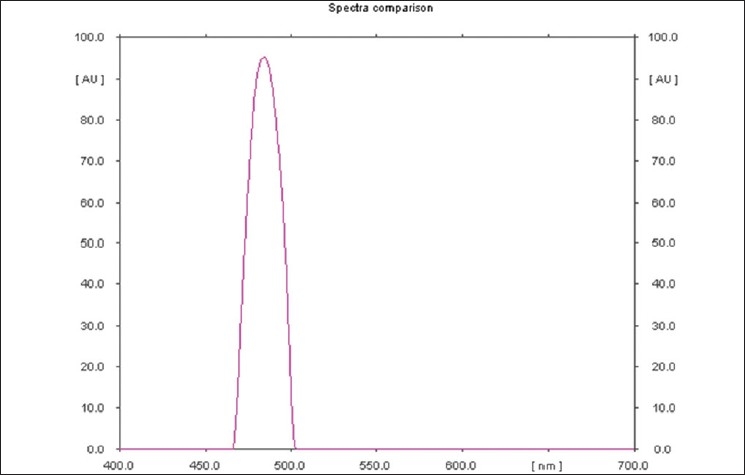
Postderivatization UV spectra of monosodium glutamate

The mobile phase resolved MSG very efficiently and is shown in Figure 1. The R_f_ value of monosodium glutamate was 0.64. Typical absorption spectra of the derivatized monosodium glutamate was shown in Figure 2. Postderivatization of monosodium glutamate with 1% ninhydrin solution gave an absorbance maximum at 485 nM and was selected for detection. The method was used to determine MSG content in three different food products. The results were tabulated in Table 1.

**Table 1:** Analysis of monosodium glutamate in food products

Sample	Amount of monosodium l-glutamate present^a^ (mg/g)
Masala	49.66 ± 1.34
Soup	24.59 ± 1.47
Cubes	133.50 ± 0.84

a RSD- Relative standard deviation of three determinations.

### System suitability

According to the USP method (621), system suitability tests are an integral part of a chromatographic analysis and should be used to verify that the resolution and reproducibility of the chromatographic system are adequate for the analysis. To ascertain the effectiveness of the method developed in this study system suitability tests were performed on freshly prepared standard stock solution of MSG.

### Linearity

Calibration plots of peak area against concentration of monosodium glutamate were linear in the range of 0.4–1.0 μg. The calibration lines were represented by the linear regression equation *Y* = 5627 + 13.08*X* where *Y* is the peak area and *X* is concentration. The correlation coefficient *r* was 0.9942.

### Limit of quantification and detection

The limit of quantification (LOQ) and limit of detection (LOD) were calculated by use of the equations LOD = 3 × *N/B* and LOQ = 10 × *N/B*, where *N* is the standard deviation of the peak area of the drug (*n* = 3), taken as a measure of the noise and *B* is the slope of the corresponding calibration curve. The limit of quantification and the limit of detection for monosodium glutamate were 0.7 and 2.3 ng, respectively.

### Accuracy and precision

The accuracy and precision of the method were studied by performing experiments by the standard addition technique. Accuracy of the method was determined by recovery experiments. The recovery of the method was determined at single level by adding a known quantity of MSG to the food products of preanalyzed samples, and the mixtures were reanalyzed according to the proposed method. The average recovery obtained for monosodium glutamate for 97.0% and is shown in Table 2.

**Table 2:** Results from recovery analysis

Amount added (mg)	Amount recovered (mg)		
	Masala	Soup	Cubes
0	49.66	24.59	133.5
2	51.68	26.65	135.58

Mean recovery (n = 3) = 102.66%.

### Ruggedness and robustness

Ruggedness is a measure of the reproducibility of a test result under normal, expected operating conditions from instrument-to-instrument and from analyst-to-analyst [Table 3]. Robustness of the method was determined by making slight changes in the chromatographic conditions. No marked changes in the chromatograms demonstrated that the high-performance thin layer chromatography (HPTLC) method developed are rugged and robust [Table 4].

**Table 3:** Results from ruggedness studies^a^

Analysts	Amount recovered (%)		
	Masala	Soup	Cubes
Analyst I	99.68	99.63	99.92
Analyst II	100.36	101.06	100.19

a All values are percentage recoveries.

**Table 4:** Results from robustness studies

Development distance (mM)	Amount recovered (mg)		
	Masala	Soup	Cubes
73.0	101.61	101.99	100.37
80.0	101.73	102.07	100.78

## CONCLUSION

The HPTLC method proposed for determination of MSG in different food products was accurate, precise, rapid, selective, and sensitive. It can, therefore, be conveniently adopted for the routine analysis of monosodium glutamate in food products.

## References

[1] (1995). U. S. Department of Health and Human Services, U. S. Food and Drug Administration. FDA and Monosodium Glutamate (MSG).

[2] Stegink LD, Filer LJ, Baker GL (1985). Plasma glutamate concentrations in adult subjects ingesting monosodium L-glutamate in consommé. Am J Clin Nutr.

[3] Meldrum B (1993). Amino acids as dietary excitotoxins: A contribution to understanding neurodegenerative disorders. Brain Res Brain Res Rev.

[4] Nemeroff C, Miller SA (1981). Monosodium glutamate-induced neurotoxicity: Review of the literature and call for further research. US FDA: Nutrition and Behavior.

[5] Acebal CC, Lista AG, Fernandez BS (2008). Simultaneous determination of flavor enhancers in stock cube samples by using spectrophotometric data and multivariate calibration. Food Chem.

[6] Durán-Merás I, Salinas F, Muñoz De La Peña A, López Rosas M (1993). Simultaneous determination of flavor enhancers inosine 5’-monophosphate and guanosine 5’-monophosphate in food preparations by derivative spectrophotometry. J AOAC Int.

[7] Populin T, Moret S, Truant S, Conte LS (2007). A survey on the presence of free glutamic acid in food stuffs, with and without added monosodium glutamate. Food Chem.

[8] Sporns P (1982). Rapid high performance liquid chromatographic determination of monosodium glutamate in food. J Assoc Off Anal Chem.

[9] Rhys Williams AT, Winfield SA (1982). Determination of monosodium glutamate in food using high-performance liquid chromatography and fluorescence detection. Analyst.

[10] Conacher HB, Iyengar JR, Miles WF, Botting HG (1979). Gas-liquid chromatographic determination of monosodium glutamate in soups and soup bases. J Assoc Off Anal Chem.

[11] Bailey BW, Swift HC (1970). A rapid paper chromatographic method for the determination of monosodium glutamate. J Assoc Off Anal Chem.

[12] Rhodes J, Titherley AC, Norman JA, Wood R, Lord DW (1991). A survey of the monosodium glutamate content of foods and an estimation of the dietary intake of monosodium glutamate. Food Addit Contam.

